# APOE4 carrier status determines association between white matter disease and grey matter atrophy in early-stage dementia

**DOI:** 10.1186/s13195-023-01251-4

**Published:** 2023-06-03

**Authors:** Ashwati Vipin, Dilip Kumar, See Ann Soo, Fatin Zahra Zailan, Yi Jin Leow, Chen Ling Koh, Adeline Su Lyn Ng, Kok Pin Ng, Nagaendran Kandiah

**Affiliations:** 1 Dementia Research Centre – Lee Kong Chian School of Medicine, Nanyang Technology University, 11 Mandalay Road, Singapore, 308232 Singapore; 2 National Neuroscience Institute, Singapore, Singapore; 3 Duke-NUS Medical School, Singapore, Singapore

**Keywords:** White matter hyperintensity, APOE4, Dementia, Cognitively normal, Grey matter, Cognition

## Abstract

**Background:**

White matter hyperintensities, a neuroimaging marker of small-vessel cerebrovascular disease and apolipoprotein ε4 (APOE4) allele, are important dementia risk factors. However, APOE4 as a key effect modifier in the relationship between white matter hyperintensities and grey matter volume needs further exploration.

**Methods:**

One hundred ninety-two early-stage dementia (including mild cognitive impairment and mild dementia) and 259 cognitively unimpaired participants from a neurocognitive research cohort with neuroimaging data, APOE genotyping, and neuropsychological assessments were studied. We investigated independent and interactive effects of white matter hyperintensities and APOE4 on whole-brain voxel-wise grey matter volume using voxel-based morphometry (uncorrected *p* < 0.001; minimum cluster size = 100 voxels). We further assessed interactive effects between APOE4 and white matter hyperintensities on global cognition, memory, and executive function in early-stage dementia and cognitively unimpaired participants.

**Results:**

Independent of APOE4 status, higher white matter hyperintensity load was associated with greater grey matter atrophy across frontal, parietal, temporal, and occipital lobes in cognitively unimpaired and early-stage dementia subjects. However, interaction analyses and independent sample analyses revealed that APOE4 non-carriers demonstrated greater white matter hyperintensity-associated grey matter atrophy compared to APOE4 carriers in both cognitively unimpaired and early-stage dementia groups. Additional confirmatory analyses among APOE4 non-carriers demonstrated that white matter hyperintensities resulted in widespread grey matter loss. Analyses of cognitive function demonstrated that higher white matter hyperintensity load was associated with worse global (Mini-Mental State Examination, Montreal Cognitive Assessment) and executive function (Color Trails 2) in APOE4 non-carriers compared to APOE4 carriers in early-stage dementia but not cognitively unimpaired participants.

**Conclusions:**

The association between white matter hyperintensities and grey matter loss is more pronounced in APOE4 non-carriers than APOE4 carriers in the cognitively unimpaired and early-stage dementia stages. Furthermore, white matter hyperintensity presence results in poorer executive function in APOE4 non-carriers compared to APOE4 carriers. This finding may have significant impact on the design of clinical trials with disease modifying therapies.

**Supplementary Information:**

The online version contains supplementary material available at 10.1186/s13195-023-01251-4.

## Introduction

Mild cognitive impairment (MCI), an intermediate stage between normal cognition and dementia, is a high risk stage for progression to dementia. Specifically, 40–60% of patients progress to dementia after 5 years, with an annual rate of progression to dementia ranging from 11 to 13% [1, 2].

Multiple risk factors predict the progression of MCI to dementia including higher age, APOE ε4 (APOE4) allele, lower educational attainment, and cerebrovascular disease (CVD) [3]. White matter hyperintensities (WMH), an MRI surrogate measure for small-vessel CVD [4], are an important risk factor for the clinical manifestation of MCI and progression to dementia [5]. In our previous work, we demonstrated a high burden of CVD among Southeast-Asian patients with MCI and dementia, with 9.7% of MCI and 28.4% of mild dementia patients harboring severe WMH burden [6]. More recently, we found that the odds ratio of patients with MCI having confluent WMH progressing to dementia was 2.66 (CI: 1.292, 5.470; *p* = 0.008) [7].

WMH has consistently been shown to have a role in grey matter (GM) structural changes and cognitive decline at early disease stages [8]. WMH-related structural GM changes involve temporal and frontal regions within the Alzheimer’s disease (AD)-related default mode and executive control networks [9]. Derogatory influences of WMH on GM volume (GMV) have also been shown to be widespread at the MCI stage, compared to both cognitively normal and dementia stages [10]. Additionally, cross-sectional studies illustrate associations between high WMH and decline in memory and executive function in healthy controls and MCI [11].

Apolipoprotein E (APOE) is involved in lipid transfer, cell metabolism, neuronal repair and amyloid-β peptide accumulation [12]. Specifically, the APOE4 allele is an established genetic risk factor that increases the incidence and lowers the age of AD onset [13]. Patients with AD having APOE4 demonstrate greater neurodegeneration [13].

While there are mixed findings from studies that evaluate the influence of APOE4 on WMH load [14, 15], structural MRI studies have demonstrated reduced GM in elderly healthy controls and MCI who are APOE4 carriers [16, 17]. In MCI and dementia, APOE4-related GM volume loss occurs in the hippocampus, amygdala, and mesial temporal cortex as well as left occipital, frontal, and anterior cingulate cortices [18]. Longitudinal studies further indicate that among MCI converters, APOE4 carriers display increased GM atrophy in AD-related brain regions [19].

While both WMH and APOE4 are independently associated with GM atrophy, recent findings also illustrate significant associations between APOE4 allele and WMH burden [20]. Additionally, a meta-analysis reported that the APOE4 allele is associated with greater burden of CVD [21]. When examining combined APOE4 and WMH effects, prior studies have demonstrated worse attention/executive function in APOE4 carriers with dementia [22]. Additionally, APOE4 status was a key moderator in the relationship between brain atrophy and functional impairment in the dementia stage [23]. However, it remains to be known if APOE4 is a key effect modifier in the relationship between WMH and GMV, especially early in the disease course.

Here, in a cohort of cognitively unimpaired (CU) and early-stage dementia (ESD, comprising participants with MCI and mild dementia), we examined the independent influence of WMH and APOE4 status on brain GMV. We further examined the moderating effect of APOE4 status on the association between WMH and GMV in CU and ESD participants. Additionally, we examined the interaction effect of APOE4 and WMH on cognitive function in ESD. We hypothesized that participants with higher WMH load would demonstrate greater GMV atrophy and greater cognitive impairment, with this effect being more pronounced in APOE4 carriers compared to APOE4 non-carriers.

## Methods

### Participants

Participants were recruited from a tertiary neurology center in Singapore between April 2013 and July 2020 for cognitive research studies investigating the association between CVD, cognition, neuroimaging characteristics, and APOE. Participants were classified as cognitively unimpaired (CU) or early-stage dementia (ESD). ESD included both MCI and mild dementia. MCI participants met NIA-AA criteria, did not have impairment in functional activity, did not meet criteria for dementia, and had a CDR of 0.5 [24]. Mild dementia patients met the NIA-AA criteria for dementia with CDR score of 1 [25]. CU participants had unimpaired functional activities, CDR of 0, and Montreal Cognitive Assessment (MoCA) score of 26 or more. Exclusion criteria included (1) history of alcohol or drug abuse, (2) current or known history of psychiatric disease such as major depression or neurological disease, (3) prior clinical stroke, and (4) presence of contraindications to MRI.

### Neuropsychological assessments

All participants, cognitively unimpaired and cognitively impaired, underwent a standardized battery of neuropsychological assessments administered by trained research staff. Cognitive information collected examined Alzheimer’s Disease Assessment Scale (ADAS)–Cognitive 10-word delayed recall [26] as a test of episodic memory function and the Color Trails 2 [27] as a test of executive function. Measures of global cognition included the Mini-Mental State Examination (MMSE) [28] and the Montreal Cognitive Assessment (MoCA) [29].

### Neuroimaging

MRI scans were performed on a 3T Prisma fit System (Siemens, Erlangen, Germany) and 3T Ingenia System (Philips Medical Systems). Each participant had high resolution T1-weighted Magnetization Prepared Rapid Gradient Echo: 192 continuous sagittal slices, TR/TE/TI = 2300/2.28/900 ms, flip angle = 8°, FOV = 256 × 240 mm^2^, matrix = 256 × 240, isotropic voxel size = 1.0 × 1.0 × 1.0 mm^3^, bandwidth = 200 Hz/pixel) and Fluid Attenuated Inversion Recovery (FLAIR) sequences (192 continuous sagittal slices, TR/TE/ TI = 5000/387.0/1800 ms, flip angle = 120°, FOV = 256 × 256 mm^2^, matrix = 256 × 256, isotropic voxel size = 1.0 × 1.0 × 1.0 mm^3^, bandwidth = 750 Hz/pixel). In the year 2018, there was a change in the 3T scanner for research participants, and the subsequent scans were performed using the 3T Ingenia System. Using the 3T Ingenia System (Philips Medical Systems), each participant had high resolution T1-weighted Magnetization Prepared Rapid Gradient Echo (180 continuous sagittal slices, TR/TE = 7.46/3.4 ms, flip angle = 8°, FOV = 256 × 256 mm^2^, matrix = 256 × 256, isotropic voxel size = 1.0 × 1.0 × 1.0 mm^3^) and FLAIR sequences (200 continuous sagittal slices, TR/TE/TI = 4800/378.15/1650 ms, flip angle = 90°, FOV = 240 × 240 mm^2^, matrix = 240 × 240, isotropic voxel size = 1.0 × 1.0 × 2.0 mm^3^). Scan images were reviewed at acquisition, and participants with motion artifacts and gross pathological findings were excluded.

### Image pre-processing

We used the Computational Anatomy Toolbox (http://dbm.neuro.uni-jena.de/cat12/) protocol in Statistical Parametric Mapping (SPM12) (http://www.fil.ion.ucl.ac.uk/spm/), to process the T1 images for voxel-based morphometry analysis. Here, all 3D T1-weighted MRI scans were normalized using an affine transformation followed by non-linear registration and corrected for bias field inhomogeneities. Images were then segmented to derive participant-level GM, white matter, and cerebrospinal fluid components [30]. The Diffeomorphic Anatomic Registration Through Exponentiated Lie algebra algorithm was used to normalize the segmented scans into the standard MNI space to provide better precision in spatial normalization to the template [31]. Subsequently, the modulation step performed a non-linear deformation on the normalized segmented images. The modulation step provides a comparison of the absolute amounts of tissue corrected for individual differences in brain size. All obtained segmented, modulated, and normalized GM images were then smoothed using an 8-mm full-width-half-maximum isotropic Gaussian smoothing kernel.

### White matter hyperintensity derivation

For WMH detection, we utilized the Lesion Segmentation Toolbox (LST version 2.0.15), a MATLAB (https://www.mathworks.com) and SPM12-based automated tool, to extract binary WMH lesion belief maps [32, 33]. The automated lesion growth algorithm from LST on T1 anatomical and FLAIR images quantified WMH as reported previously [34]. This algorithm first co-registers the T2 FLAIR to T1 and subsequently segments T1 images into GM, white matter, and cerebrospinal fluid tissue maps. This information is then combined with co-registered T2 FLAIR images to estimate the WMH lesion belief maps. By thresholding these maps with a pre-determined initial kappa threshold (κ), an initial binary lesion map is obtained and is subsequently grown along voxels that appear hyperintense on the T2 FLAIR image. To define this optimal threshold, T1 and FLAIR images of 10 randomly chosen participants with mild to severe WMH load were segmented at *κ* = 0.30, *κ* = 0.20, and *κ* = 0.10. After further visual inspection of segmentation results at these threshold levels, the WMH visual raters determined *κ* = 0.10 as the optimal threshold. The total lesion volume in each participant was then obtained using the “extract values of interest” option in the LST toolbox.

In the following sections, WMH load will refer to log-transformed WMH. Additionally, lesion probability maps generated by LST were used on T1-weighted images for lesion filling to correct for presence of white matter lesions which may lower the estimated GM fraction [32]. These lesion-filled images were used for subsequent group analyses.

### Apolipoprotein E genotyping

Genomic DNA was extracted from peripheral blood using QIAamp® DNA Blood Maxi Kit (Qiagen GmbH, Hilden, Germany) as per standard protocol. Genotyping for APOE isoforms [rs429358 (ABI assay ID:C_3084793_20) was performed using TaqMan SNP genotyping assays on ABI 7900HT PCR system (Applied Biosystems, Foster City, CA). APOE genotype assignments were performed as described [35].

### Statistical analyses

Group differences on participant characteristics between the CU and ESD group were assessed using two-sample *t*-test analyses for continuous variables and chi-square tests for categorical variables (Table 1).

**Table 1:** Participant demographics

	**Cognitively unimpaired (*n* = 259)**	**Early-stage dementia (*n* = 192)**	***P*-value**
**Age, years, mean (SD)**	61.83 (8.2)	65.16 (9.28)	< 0.001
**Female, *n* (%)**	140/259 (54.1%)	102/192 (53.1%)	0.845
**Education, years, mean (SD)**	13.05 (3.73)	10.29 (3.73)	< 0.001
**APOE4 carrier, *n* (%)**	33/259 (12.7%)	61/192 (31.8%)	< 0.001
**Total GMV, mean (SD)**	579.15 (54.3)	555.59 (58.9)	< 0.001
**Total ICV, mean (SD)**	1356.23 (146.5)	1357.83 (143.1)	0.908
**WMH Volume, mean (SD)**	6.87 (9.60)	9.46 (12.81)	0.015
**MMSE, mean (SD)**	28.56 (1.28)	25.14 (3.18)	< 0.001
**MoCA, mean (SD)**	27.80 (1.23)	21.57 (2.85)	< 0.001

*Abbreviations*: *APOE4* Apolipoprotein epsilon 4, *GMV* Grey matter volume, *ICV* Intracranial volume, *WMH* White matter hyperintensity, *MMSE* Mini-mental state examination, *MoCA* Montreal cognitive assessment

#### Voxel-based morphometry analyses

To assess whether the variance in GMV values obtained was different between the two scanners, we derived GMV from representative regions-of-interest (ROIs) in the frontal, temporal, and parietal lobes using the Automated Anatomical Labelling atlas. We then carried out an *F*-test to assess if the variance of GMV in these ROIs was different between the two scanners. We also carried out non-parametric Kolmogorov–Smirnov tests to assess the null hypothesis that the ROI GMV values from the two scanners were drawn from the same continuous distribution.

We assessed differences in grey matter atrophy between the CU and ESD group and found significantly greater atrophy in the ESD group. Thus, all analyses were carried out separately in CU and ESD participants to assess if there was a stage-dependent effect of WMH on GMV and whether this effect was different among APOE4 carriers and non-carriers.

##### Association between WMH, APOE4 status and voxel-wise GMV

To assess the positive and negative effect of WMH, APOE4 status on voxel-wise GMV, we built a voxel-wise multiple regression model with GMV as the dependent variable and log-transformed WMH or APOE4 status as the independent variable of interest. Age, sex, years of education, total intracranial volume, and scanner type were added as covariates. When log-transformed WMH was the independent variable of interest, APOE4 was also included as a covariate in the analyses and vice versa. The effects of WMH and APOE4 status were examined in different models and conducted separately for the CU and ESD participants.

##### Interaction effect of WMH and APOE4 status on voxel-wise GMV

To assess the effect of WMH on voxel-wise GMV between APOE4 carriers and non-carriers, we built a voxel-wise multiple regression model with GMV as the dependent variable and log-transformed WMH * APOE4 status interaction term as the independent variable of interest. Age, sex, years of education, total intracranial volume, and scanner were added as covariates. This analysis was conducted separately for the CU and ESD participants.

##### Confirmatory analysis of the effect of WMH on voxel-wise GMV in APOE4 non-carriers and carriers

To assess effect of WMH on voxel-wise GMV in APOE4 non-carriers and carriers, we also built a voxel-wise multiple regression model with GMV as the dependent variable and log-transformed WMH as the independent variable of interest. Age, sex, years of education, total intracranial volume, and scanner were added as covariates. This analysis was conducted separately for the CU and ESD APOE4 non-carriers and carriers.

The GM clusters showing significant main and interaction effects were examined using a threshold of uncorrected *p* < 0.001 and a minimum cluster size of 100 voxels [10]. Significant GM clusters were identified using the Automated Anatomical Labelling atlas.

#### Interaction effect of WMH and APOE4 status on cognition

To assess the interaction effect of WMH load and APOE4 status on cognition, we carried out separate linear regression analyses with MMSE, MoCA, ADAS delayed-recall, and Color Trails 2 scores as the dependent variable and log-transformed WMH*APOE4 status interaction term as the independent variable of interest. Age, sex, years of education, total intracranial volume, and scanner were added as covariates to the model. This analysis was performed separately in the CU and ESD groups.

All statistical analyses were performed using R 3.6.3 (R Core Team, 2014) with RStudio (RStudio Team, 2012).

## Results

### Demographics

A total of 451 participants comprising 192 ESD and 259 CU participants were included in the study. Of the 192 ESD participants, 124 had a diagnosis of MCI and the remaining 68 had a diagnosis of mild dementia. Participant demographics are detailed in Table 1. ESD participants were significantly older at diagnosis, had fewer years of education, had higher frequency of APOE4 carriers, and had lower total GMV and overall higher WMH volume, corrected for age, sex, education years, and total intracranial volume.

### White matter hyperintensity volume is associated with widespread voxel-wise grey matter atrophy

*F*-test analyses revealed no differences in the variance of GMV values between the two scanners for the superior medial frontal cortex, precuneus, and hippocampus ROIs (*p* > 0.05). Kolmogorov–Smirnov tests also illustrated that the ROI GMV values for the superior medial frontal cortex, precuneus, and hippocampus obtained from the two scanners came from a similar distribution (*p* > 0.05). Nonetheless, we additionally controlled for scanner type in all our analyses.

**Fig. 1:**
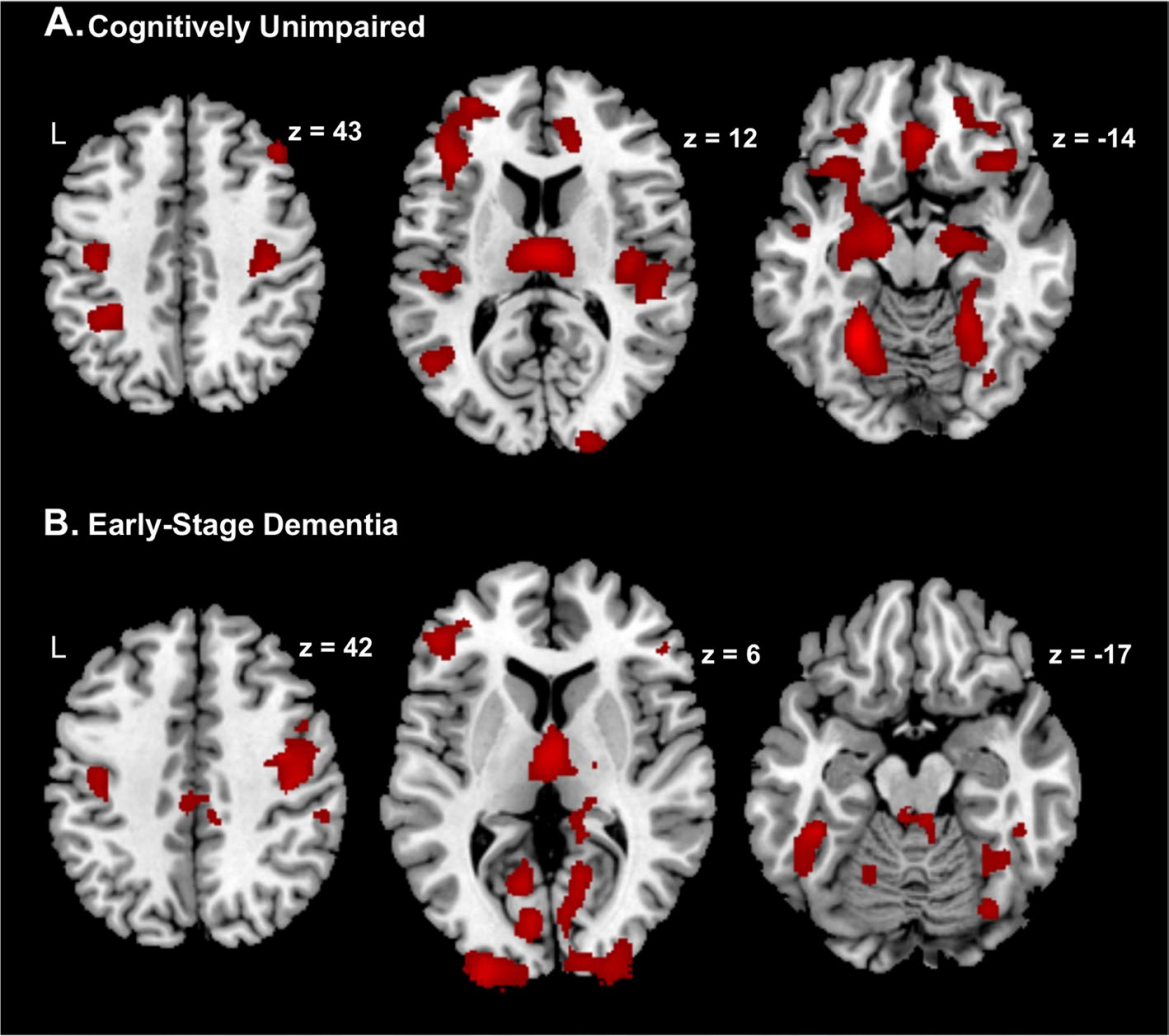
Negative influence of white matter hyperintensity load on voxel-wise grey matter volume in cognitively unimpaired and early-stage dementia participants. In both cognitively unimpaired (A) and early dementia participants (B), increasing WMH load was associated with lower voxel-wise GMV across frontal, parietal, temporal, and occipital regions. Clusters showing GMV loss related to WMH are shown in red. There were no positive associations between WMH load and voxel-wise GMV. Results are shown at the uncorrected *p* < 0.001 height threshold with an extent threshold of 100 voxels. Results are displayed on representative sections of the MNI template brain. WMH, white matter hyperintensity; GMV, grey matter volume

In CU participants, higher WMH load was associated with more widespread voxel-wise GMV loss across the frontal, parietal, temporal, and occipital lobes. Regions of lower GMV included the bilateral medial orbitofrontal, middle and inferior frontal cortex, bilateral anterior cingulate cortex, bilateral middle and superior temporal cortex, bilateral hippocampal and parahippocampal cortex, bilateral insula, bilateral precentral and postcentral gyrus, and middle and posterior cingulate cortex as well as right middle and superior occipital cortex and right middle and superior occipital cortex (uncorrected *p* < 0.001; minimum cluster size = 100 voxels; Fig. 1A).

In ESD participants, higher WMH load was associated with more voxel-wise GMV loss within the parietal, occipital, and temporal regions. Regions of lower GMV included bilateral superior, middle and inferior occipital regions, bilateral precentral and postcentral gyrus, bilateral precuneus, bilateral middle and inferior temporal regions, and some parts of the middle and inferior frontal cortex (uncorrected *p* < 0.001; minimum cluster size = 100 voxels; Fig. 1B).

### White matter hyperintensity load is associated with more widespread voxel-wise grey matter atrophy in APOE4 non-carriers compared to APOE4 carriers

**Fig. 2:**
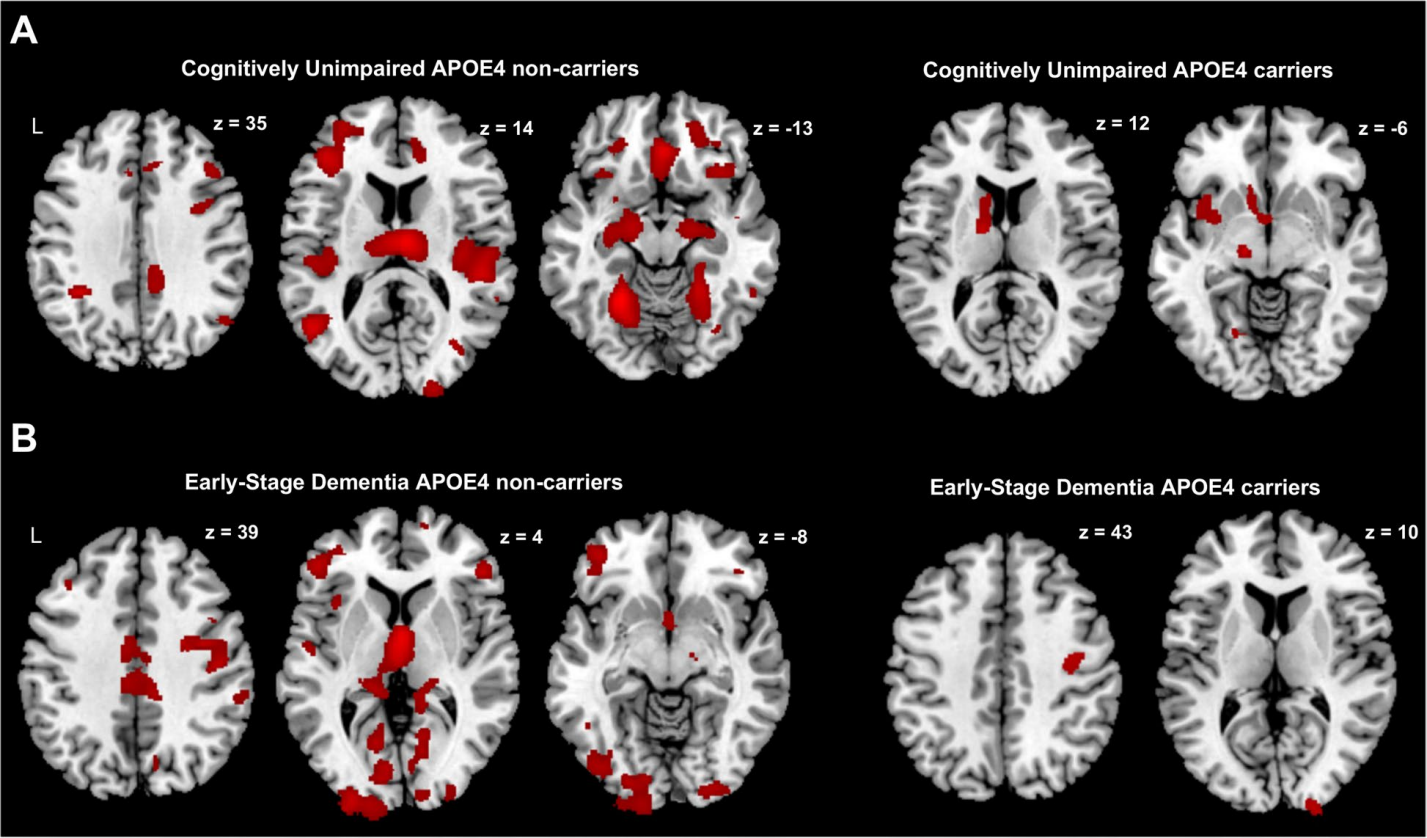
Among cognitively unimpaired and early-stage dementia participants, APOE4 carrier status influences the effect of white matter hyperintensity load on voxel-wise grey matter volume. In both cognitively unimpaired (**A**) and early-stage dementia participants (**B**) APOE4 non-carriers, increasing WMH load was associated with widespread lower voxel-wise GMV across frontal, parietal, temporal, and occipital regions only in APOE4 non-carriers. On the other hand, in cognitively unimpaired (**A**) APOE4 carriers, increasing WMH load was associated with lower voxel-wise GMV only in the left middle and superior temporal pole, caudate and lingual gyrus and in early-stage dementia participants (**B**) only in right precentral and superior occipital gyrus. Clusters showing GMV loss related to WMH are shown in red. Results are shown at the uncorrected *p* < 0.001 height threshold with an extent threshold of 100 voxels. Results are displayed on representative sections of the MNI template brain. WMH, white matter hyperintensity; GMV, grey matter volume; APOE4, apolipoprotein E4

When assessing the interaction effect between WMH load and APOE4 status, higher WMH load was associated with more widespread GMV loss across frontal, parietal, temporal, and occipital lobes, among CU APOE4 non-carriers, Regions showing negative associations between WMH load and GMV among APOE4 non-carriers included bilateral hippocampal and parahippocampal regions, bilateral thalamus, bilateral middle and superior temporal cortex, bilateral superior, middle and inferior occipital cortex, right postcentral and superior parietal cortex and bilateral middle, and inferior frontal and medial orbitofrontal cortices (uncorrected *p* < 0.001; minimum cluster size = 100 voxels; Fig. 2A). In the CU APOE4 carriers, higher WMH load was associated with fewer regions of GMV loss including the left middle and superior temporal pole, left caudate, and left lingual gyrus.

Similarly, among ESD APOE4 non-carriers, higher WMH load was associated with more widespread GMV loss across frontal, temporal, and occipital lobes. Regions showing negative associations between WMH load and GMV included bilateral superior, middle and inferior frontal cortex, bilateral precentral and postcentral gyrus, bilateral middle cingulate, bilateral superior and middle temporal cortex, bilateral superior, and middle and inferior occipital cortex and bilateral thalamus (*p* < 0.001; minimum cluster size = 100 voxels; Fig. 2B). In the ESD APOE4 carriers, higher WMH load was associated with GMV loss only in the right precentral gyrus.

We also carried the same analyses as above using an FDR-corrected threshold of *p* < 0.05 and extent threshold of 100 voxels, and all results remained. All these associations were observed despite no baseline differences in WMH load between APOE4 carriers and non-carriers.

In the additional confirmatory analyses on WMH effects among APOE4 non-carriers, we observed the same regions showing widespread GMV loss across frontal, temporal, parietal, and occipital lobes related to higher WMH load (uncorrected *p* < 0.001; minimum cluster size = 100 voxels) as observed in the interaction effect analyses. This overlap in WMH effect on GMV in APOE4 non-carriers was observed at both the CU and ESD stages. For the confirmatory analyses on WMH effects among APOE4 carriers, we observed similarly few regions showing GMV loss related to higher WMH load in the bilateral caudate and left putamen regions in the CU group and in the right precentral gyrus and left middle superior frontal gyrus in the ESD group (uncorrected *p* < 0.001; minimum cluster size = 100 voxels).

### Effects of APOE4 status on voxel-wise grey matter volume

In the CU, APOE4 carriers had lower voxel-wise GMV in the left angular gyrus, left inferior and middle temporal cortex, right postcentral and precentral gyrus, and left inferior parietal cortex and left inferior frontal cortex compared to APOE4 non-carriers (uncorrected *p* < 0.001; minimum cluster size = 100 voxels). On the other hand, APOE4 non-carriers did not show reduced GMV compared to APOE4 carriers in any region in the CU.

In the ESD, APOE4 carriers had lower voxel-wise GMV in the left inferior parietal cortex and bilateral hippocampus compared to APOE4 non-carriers (uncorrected *p* < 0.001; minimum cluster size = 100 voxels). Additionally, APOE4 non-carriers showed lower GMV primarily in the right middle frontal cortex than APOE4 carriers.

### White matter hyperintensity load among early-stage dementia APOE4 non-carriers relates to worse cognition

**Fig. 3:**
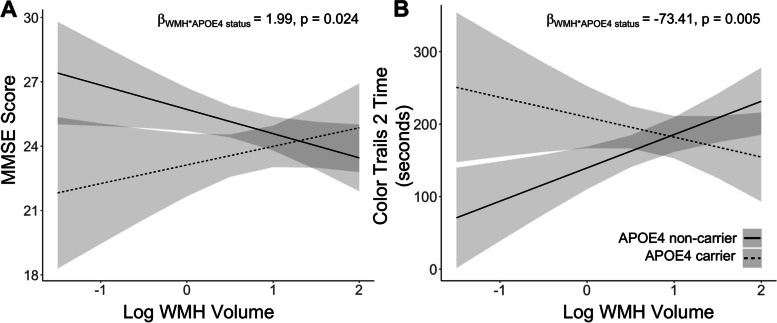
White matter hyperintensity load among early-stage dementia APOE4 non-carriers relates to worse cognition than APOE4 carriers. In early-stage dementia participants, there was a significant interaction effect between WMH load and APOE4 status on cognition. Increasing white matter hyperintensity load was associated with (**A)** lower MMSE scores and (**B)** poorer Color Trails 2 performance in APOE4 non-carriers compared to APOE4 carriers. The solid line refers to APOE4 non-carriers while the dashed line refers to APOE4 carriers. APOE4, apolipoprotein E4; MMSE, mini-mental state examination; WMH, white matter hyperintensity

In ESD participants, we assessed whether the interaction between WMH and APOE4 status resulted in worse global and domain-specific cognition. Specifically, in APOE4 non-carriers, a linear regression analyses illustrated that higher WMH load was associated with worse global cognition on the MMSE (*β*_WMH*APOE4 status_ = 1.99, *p* = 0.024, CI 0.25:3.74; Fig. 3A, Supplementary Fig. 1A), MoCA (*β*_WMH*APOE4 status_ = 2.31, *p* = 0.0047, CI 0.71:3.90) and executive function on the Color Trails 2 (*β*_WMH*APOE4 status_ =  − 73.41, *p* = 0.005, CI − 124.61: − 22.22; Fig. 3B, Supplementary Fig. 1B). These results were controlled for age, sex, education years, and scanner type. Additionally, there was no interaction effect of WMH and APOE4 on memory using the ADAS-delayed recall (*β*_WMH*APOE4 status_ =  − 1.23, *p* = 0.139, CI -2.86:0.40).

On the other hand, in CU participants, WMH and APOE4 status did not influence global (MMSE, MoCA) or domain-specific cognition (ADAS-delayed recall, Color Trails 2). Thus, there was no significant interaction effect of WMH and APOE4 status on cognitive performance in the CU group.

## Discussion

In the present study, we examined both independent effects of WMH and APOE4 as well as interactive effects of WMH and APOE4 on whole-brain voxel-wise GMV in CU and ESD participants. We show that higher WMH is associated with more widespread GM atrophy across frontal, parietal, temporal, and occipital lobes in both CU and ESD. However, when examining the interaction effect of WMH and APOE4 on GMV, we demonstrate that contrary to our hypothesis, the association between WMH and GMV loss was greater in non-APOE4 carriers compared to APOE4 carriers. This interaction effect also extended into cognitive performance with APOE4 non-carriers showing worse global and executive function in the presence of high WMH load compared to APOE4 carriers at the ESD stage. These associations were observed despite no baseline differences in WMH load between APOE4 carriers and non-carriers. Our findings suggest differential effects of WMH on GMV and cognition depending on the APOE4 status.

Our findings illustrate widespread negative effects of WMH on voxel-wise GMV in both CU and ESD participants. In this regard, higher WMH load was associated with greater GMV loss in frontal, parietal, temporal, and occipital regions in CU and in parietal, temporal, and occipital regions in ESD. Such derogatory WMH effects on GMV are consistent with previous findings of widespread GM loss, especially in cognitively normal middle-aged and elderly as well as early-stage dementia participants [10, 36]. While the exact mechanisms through which WMH result in GMV loss is not known, postulated processes include Wallerian neurodegeneration of the axons resulting in neuronal loss and small vessel ischemia causing both WMH and neuronal loss as well as neuroinflammation induced WMH and neuronal loss [37–39].

Consistent with previous work, APOE4 carriers in our study showed GMV loss in CU and ESD participants in parietal and temporal regions compared to APOE4 non-carriers [17]. Thus, our findings further confirm that the APOE4 allele contributes to GM atrophy even in pre-clinical and prodromal stages of disease [17]. The postulated mechanisms by which APOE4 results in neurodegeneration include amyloid-β dependent and non-amyloid related mechanisms. Specifically for amyloid-β, APOE4 is thought to exacerbate oligomeric amyloid-beta associated neurodegeneration involving intraneuronal uptake of soluble amyloid-beta, in turn leading to mitochondrial impairment as well as extracellular effects leading to synaptic loss [40]. Non-amyloid mechanisms of APOE-induced neurodegeneration comprise tau-mediated neuronal death and alterations in synaptic morphology as well as dysregulation of calcium homeostasis with associated cell death [41]. Prior studies also show differential effects regarding how APOE4 status modulates the relationship between amyloid-beta pathology, cerebrovascular disease, and neurodegeneration. Some show no influence of cerebrovascular disease on the association between APOE4 and amyloid-beta [42]. However, in support of our findings, one notable study illustrated that larger WMH burden related to faster decreases in hippocampal volume and cognition in APOE4 non-carriers [43]. Thus, future studies that assess biomarker profiles in APOE4 non-carriers will be imperative in providing further insights into these links [44].

In this study, we demonstrate derogatory effects of WMH on GMV loss predominantly in APOE4 non-carriers. This was true for both the interaction between WMH and APOE4 status as well as on examination of the WMH effect on GMV in APOE4 non-carriers only. In this regard, previous studies in AD patients indicate that significantly greater WMH burden in APOE4 non-carriers than APOE4 carriers, although not the case in our cohort, may constitute a possible underlying factor leading to greater GMV loss in APOE4 non-carriers [45]. Recent studies have also shown disparate relationships between AD pathologies involving amyloid-beta and tau and markers of neuroinflammation as well as microglia in APOE4 carriers and non-carriers [46]. Specifically, levels of inflammatory markers such as interleukin-4 and interleukin-10 negatively associated with AD pathology in APOE4 non-carriers [46]. Furthermore, pro-inflammatory inflammasome activation involving interleukin secretion has also been illustrated in APOE4 non-carriers, subsequently promoting atherosclerosis [47]. Additionally, APOE4-related blood brain barrier disruptions through effects on endothelial dysfunction, pericyte degeneration, and neuroinflammation may also lead to WMH development and further brain structure disruptions [15, 48]. However, APOE4 non-carriers also appear to be prone to microvascular hemorrhages and vascular frailty which may reflect increased blood brain barrier dysfunction among E4 non-carriers [15, 48]. Thus, various neuroinflammatory processes may underlie some of the differential GM changes we observe between APOE4 carriers and non-carriers. Additionally, amyloid-beta burden in posterior brain regions has been shown to be associated with WMH especially in APOE4 non-carriers, which may result in GM atrophy [49]. In the present study, GM effects in APOE4 non-carriers were present in both CU and ESD, despite the ESD group having significantly greater proportion of APOE4 carriers. The earlier findings in conjunction with our findings of greater derogatory effects of WMH on GMV in APOE4 non-carriers lend evidence to possible overriding effects of the APOE4 allele in carriers. On the other hand, CVD burden including WMH is likely to be the major influencer on pathology, brain structure, and cognition in APOE4 non-carriers. Thus, APOE4 non-carriers may have upstream mechanisms involving the CVD and neuroinflammatory pathways rather than the AD amyloid-beta pathway. This will need to be examined in further detail in future studies.

When examining the differential effects of APOE4 status on the association between WMH and cognition, higher WMH load was associated with worse MMSE, MoCA, and Color Trails 2 performance in ESD APOE4 non-carriers than APOE4 carriers. Thus, WMH and APOE4 interactive effects on GMV were also reflected in cognitive performance. The Color Trails 2 is an important component of executive function and prior studies have indicated WMH associations with executive function [50]. Indeed, prior studies have illustrated moderating effects of APOE4 on the association between WMH and cognitive performance [22, 51]. However, while earlier studies have shown worse cognition associated with WMH in APOE4 carriers, interestingly, associations between WMH and cognition were observed primarily in APOE4 non-carriers in our study. Notably, we observed worse cognition in relation to WMH in non-carriers despite no difference in WMH volume between carriers and non-carriers in ESD. In support of our findings, similar associations between WMH and cognition in APOE4 non-carriers have been observed [45]. In this regard, the apparent disparity in findings regarding the effects of APOE4 status in our study and others could be due to widespread white matter injury and CVD-related effects on brain structure in APOE4 non-carriers rather than susceptibility to more amyloid-induced damage in APOE4 carriers [45]. While not the case in our study, APOE4 non-carriers have been shown to harbor greater WMH burden [45]. Indeed, some evidence emphasizes a different set of complex factors likely confer vulnerability to microvascular damage in APOE4 non-carriers [52]. However, whether such associations between small vessel disease and cognitive impairment are indeed stronger in APOE4 non-carriers will need to be further examined across a wider spectrum of cognitive domains in future longitudinal studies.

## Limitations

Our study has several limitations. Our analyses are based on cross-sectional data across two scanners; these findings need to be further validated using a longitudinal dataset with a more comprehensive neuropsychological test battery. Moreover, participants' amyloid-beta, tau, and neuroinflammatory status were unknown, and future biomarker supported studies will provide greater insights to the underlying mechanisms of WMH and APOE4 interactions.

## Conclusions

In summary, our study demonstrates widespread WMH effects on whole-brain voxel-wise GMV in both CU and ESD stages. Notably, our study illustrates that APOE4 status is likely a key moderator in the relationship between WMH and GMV not only in the CU stage but also in ESD. Additionally, APOE4 also appears to be an important moderator in the relationship between WMH and executive function, a key domain affected in CVD especially in ESD. With APOE4 non-carriers showing greater GM atrophy as well as worse cognitive performance compared to APOE4 carriers, our findings suggest that different upstream pathways that lead to grey matter atrophy are invoked by WMH depending on the APOE4 status. Future biomarker supported longitudinal studies should assess how WMH and APOE4 interact to influence brain structure and function across the dementia spectrum.

## Supplementary Information

**Additional file 1: Supplementary Figure 1.** White matter hyperintensity load among early-stage dementia APOE4 non-carriers relates to worse cognition than APOE4 carriers. In early-stage dementia participants, there was a significant interaction effect between WMH load and APOE4 status on cognition. Increasing white matter hyperintensity load was associated with (A) lower MMSE scores and (B) poorer Color Trails 2 performance in APOE4 non-carriers compared to APOE4 carriers. The solid line refers to APOE4 non-carriers while the dashed line refers to APOE4 carriers. Abbreviations: APOE4, apolipoprotein E4; MMSE, mini-mental state examination; WMH, white matter hyperintensity.

## Data Availability

Anonymized data will be shared by request from any qualified investigator.

## Abbreviations

APOE4: Apolipoprotein ε4
WMH: White matter hyperintensity
CU: Cognitively unimpaired
ESD: Early-stage dementia
GM: Grey matter
GMV: Grey matter volume
MCI: Mild cognitive impairment
CVD: Cerebrovascular disease
ADAS: Alzheimer’s Disease Assessment Scale
ROI: Region of interest
LST: Lesion segmentation toolbox
