# Supplementary material for: APOE4 carrier status determines association between white matter disease and grey matter atrophy in early-stage dementia
Source: Alzheimers Res Ther. 2023 Jun 3;15:103. doi: 10.1186/s13195-023-01251-4 (PMC10239168; doi:10.1186/s13195-023-01251-4)
Supplement: Supplementary file 1 — Additional file 1: Supplementary Figure 1. White matter hyperintensity load among early-stage dementia APOE4 non-carriers relates to worse cognition than APOE4 carriers. In early-stage dementia participants, there was a significant interaction effect between WMH load and APOE4 status on cognition. Increasing white matter hyperintensity load was associated with (A) lower MMSE scores and (B) poorer Color Trails 2 performance in APOE4 non-carriers compared to APOE4 carriers. The solid line refers to APOE4 non-carriers while the dashed line refers to APOE4 carriers. Abbreviations: APOE4, apolipoprotein E4; MMSE, mini-mental state examination; WMH, white matter hyperintensity. [file 13195_2023_1251_MOESM1_ESM.docx]

**SUPPLEMENTARY RESULTS**

**Supplementary Figure 1**

**White matter hyperintensity load among early-stage dementia APOE4 non-carriers relates to worse cognition than APOE4 carriers.**

In early-stage dementia participants, there was a significant interaction effect between WMH load and APOE4 status on cognition. Increasing white matter hyperintensity load was associated with (A) lower MMSE scores and (B) poorer Color Trails 2 performance in APOE4 non-carriers compared to APOE4 carriers. The solid line refers to APOE4 non-carriers while the dashed line refers to APOE4 carriers.

Abbreviations: APOE4, apolipoprotein E4; MMSE, mini-mental state examination; WMH, white matter hyperintensity.
